# Overexpression of ERG and Wild-Type PTEN Are Associated with Favorable Clinical Prognosis and Low Biochemical Recurrence in Prostate Cancer

**DOI:** 10.1371/journal.pone.0122498

**Published:** 2015-04-21

**Authors:** Sung Han Kim, Soo Hee Kim, Jae Young Joung, Geon Kook Lee, Eun Kyung Hong, Kyung Min Kang, Ami Yu, Byung Ho Nam, Jinsoo Chung, Ho Kyung Seo, Weon Seo Park, Kang Hyun Lee

**Affiliations:** 1 Department of Urology, Center for Prostate Cancer, Research Institute and Hospital of National Cancer Center, Goyang, Gyeonggi-do, Korea; 2 Department of Pathology, Yonsei University Severance Hospital, Seoul; 3 Department of Pathology, Center for Prostate cancer, Research Institute and Hospital of National Cancer Center, Goyang, Gyeonggi-do, Korea; 4 Biometric Research Branch, National Cancer Center, Goyang, Gyeonggi-do, Korea; 5 Department of Cancer Control and Policy, Graduate School of Cancer Science and Policy, National Cancer Center, Goyang, Gyeonggi-do, Korea; IPO, Portuguese Institute of Oncology of Porto, PORTUGAL

## Abstract

**Objectives:**

The aim of this study was to investigate the expression of two commonly altered genes *ERG* and *PTEN* in prostate cancer (PC) and evaluate their prognostic significance. Despite conflicting published results, TMPRSS2-ERG gene fusion and PTEN loss are generally considered unfavorable markers for PC progression.

**Materials and Methods:**

Of the 762 prostatic adenocarcinoma specimens obtained from radical prostatectomy, 613 without neoadjuvant hormone therapy were included in tissue microarrays for quantitatively assessment of ERG and PTEN expression via immunohistochemistry. Statistical analysis of the association between such expression and clinicopathological parameters, including clinical prognosis, was performed with a p-value of <0.05 considered significant.

**Results:**

During a median follow-up period of 44.0 months, 132 (21.5%) patients developed biochemical recurrence (BCR). ERG overexpression and PTEN loss were observed in 145 (23.7%) and 253 (41.3%) cases, respectively. BCR-free survival was significantly better in patients with ERG overexpression (p=0.005), but unfavorable among those with PTEN loss (p=0.142). Sub-group analysis revealed that patients with PTEN loss and negative ERG expression had the worst BCR-free survival outcome (p=0.021). Furthermore, multivariate analysis identified prostate-specific antigen level (≥10 ng/mL), Gleason score (>6), pathologic T stage (≥T3), positive surgical margin, and extraprostatic capsule extension as significant risk factors for BCR (p<0.05).

**Conclusions:**

Our results indicated that ERG overexpression was associated with favorable BCR-free survival after radical prostatectomy for PC, whereas PTEN loss was with unfavorable outcomes.

## Introduction

Prostate cancer (PC), the most prevalent cancer in men, is the second leading cause of male cancer death, not only in Western countries but also in the Asia [1]. Despite diverse multimodality treatment options and extensive researches, PC remains a major health burden in men, and its diverse clinical outcomes regarding progression is a problem to be addressed owing to the disease’s heterogeneity. Such a challenging diversity necessitates the proper stratification of patients according to risk factors, such as levels of prostate-specific antigen (PSA) and its derivatives, Gleason score, and disease stage. However, these factors are not perfect; therefore, other validating tools have been investigated to distinguish important molecular basis for better prognostic prediction of PC.

In recent years, genetic aberrations, such as chromosomal translocations, have been reported in most PC cases [2]. Previous studies have identified the TMPRSS2 (androgen-regulated transmembrane protease serine 2)-ERG gene fusion on chromosome 21 as the most common aberration and an important key driver in PC [3, 4]. Such an event juxtaposes the androgen-responsive TMPRSS2 gene promoter to the coding region of the oncogenic ETS family transcription factor ERG as a result of double-strand DNA break and improper repair induced by androgen and/or genomic stress [5], subsequently leading to abnormally high expression of ERG protein. As TMPRSS2-ERG gene fusion is highly specific and arises as an early molecular event in PC [6], its association to clinical and pathological parameters has been extensively studied to evaluate its potential as a PC diagnostic and prognostic predicting tool. However, the role of ERG in PC prognosis remains debatable to date, mostly owing to different reported clinical outcomes [7–10].

Another commonly observed genomic event associated with the prognosis of human PC is PTEN (phosphatase tension homolog) genomic deletion [11–13]. PTEN loss has also been identified as one of the most common concomitant events with ERG genomic rearrangement and an important negative regulator of the PI3K/AKT signaling pathway [13,14]. Recent studies on PTEN loss and ERG rearrangement have indicated a possible association between the genetic alteration events and unfavorable clinical outcome measures [4, 11, 13].

The aim of this study was to assess the expression profiles of ERG and PTEN in Korean patients with PC via immunohistochemical (IHC) analysis of specimens obtained from radical prostatectomy and to evaluate their correlation to clinicopathological variables or prognostic characteristics of progression-free survival.

## Materials and Methods

### Patients and tissue samples

From February 2005 to December 2013, 762 consecutive PC patients who underwent radical prostatectomy at the Center for Prostate Cancer, National Cancer Center, Korea were prospectively identified. Of these, 613 cases were retrospectively reviewed after patients with missing information such as loss to follow-up or follow-up duration of less than a year, those who did not reach a postoperative undetectable PSA level of <0.2 ng/mL, and those with a history of neoadjuvant hormone therapy were excluded. All cases were independently reviewed by two pathologists (Dr. WSP and LGW) according to the guidelines of the 2005 International Society of Urological Pathology (ISUP) consensus conference [15]. All final prostatectomy specimens were also reviewed again by two pathologists (Drs. WSP and SHK). Clinical data were obtained from patients’ medical records. All study protocols were conducted according to the ethical guidelines of the “World Medical Association Declaration of Helsinki-Ethical Principles for Medical Research Involving Human Subjects.” This study was approved by the Institutional Review Board of the Research Institute and Hospital National Cancer Center (IRB No. NCCNCS 05–049). All enrolled patients provided written informed consent.

### Construction of tissue microarray

Tissue microarray (16) blocks of representative tumor areas and paired normal tissue samples were manufactured as previously described [16]. Duplicates of core tissues (2 mm in diameter) were obtained from individual donor blocks and arranged in new recipient TMA paraffin blocks using a trephine apparatus (SuperBioChips Laboratories, Seoul, Korea) [17]. All TMA blocks were confirmed to contain suitable tumor and normal tissues via hematoxylin and eosin staining. A total of 37 TMA blocks were created from this patient cohort.

### Immunohistochemical analysis and assessment

IHC analysis of ERG and PTEN expression was performed on 4-μm sections from TMA blocks using a Ventana automatic immunostainer (Ventana, Benchmark, Tuscan, AZ) and following a standard protocol. Primary antibodies used in this study were ERG (1:100, EPR 3864, Epitomics, Burlingame, CA, USA) and PTEN (1:400, 28H6, Novocastra Laboratories Ltd, Newcastle on Tyne, UK). After deparaffinization, heat-induced antigen retrieval of ERG antibodies was performed in pH 8.0 EDTA buffer as per the manufacturer’s instructions (CC1 protocol, Ventana). For PTEN, freshly cut TMA sections were deparaffinized and incubated in pH 8.0 EDTA-citrate buffer at 98°C in a microwave for antigen retrieval as per the manufacturer’s instructions. Reactivity was detected using an I-View detection kit (Ventana Medical System).

We analyzed the intensity and extent of ERG immunostaining. The nuclei of endothelial cells were used as an intrinsic positive control for ERG protein expression. The immunostaining results were analyzed semi-qualitatively for ERG. The staining intensity was categorized using a four-tiered system as negative (0, no staining), weak (+1, only visible at high magnification), moderate (+2, visible at low magnification), or strong (+3, remarkable at low magnification). The staining extent was evaluated as the fraction of positive tumor cells for each tissue spot. A final score was determined from these two parameters as follows: negative (0), absence of ERG staining in 100% of tumor cells; weak (1), intensity of 1+ in >70% of tumor cells or staining intensity of 2+ in ≤30% of tumor cells; moderate (2), intensity of 1+ in >70% of tumor cells, or staining intensity of 2+ in >30% but ≤ 70% of tumor cells, or staining intensity of 3+ in ≤30% of tumor cells; strong (3), intensity of 2+ in >70% of tumor cells, or staining intensity of 3+ in >30% of tumor cells [13]. The negative (0) and weak (1) samples were considered as negative ERG expression, whereas those with moderate (2) or strong (3) scores as positive ERG expression.

PTEN immunoreactivity was examined via the comparison of staining intensity between PC specimens and normal tissue. The IHC staining intensity was judged either normal or reduced as compared to PTEN expression on positive and negative control samples. Nuclear staining intensity of PTEN was estimated as follows: 0, negative (no appearance of stained cells); 1+, weak (0–25% of all cells were positively stained); 2+, moderate (25–50% of all cells were positively stained); and 3+, strong (>50% of all cells were positively stained). Disappearance of more than 25% of stained cells (intensity 0 and 1+) was defined as an absence of PTEN expression [11]. Interpretation of all immunostaining results was evaluated independently. In the rare instance of discrepancy, a consensus was reached via discussion on multi-head microscopic observations.

### Statistical analysis

Student *t*-test, Pearson’s λ^2^ test, or Fisher’s exact test was used for the comparison of differences in recurrence rate and various clinicopathological variables among patient groups. In this study, disease progression was defined as biochemical recurrence (BCR) after prostatectomy, which was defined as a postoperative serum PSA elevation of >0.2 ng/mL assessed on two different occasions following a decrease to non-detectable levels [18]. The first PSA value of 0.2 ng/mL or greater was used to define the time of recurrence. BCR-free survival curves were plotted using the Kaplan-Meier method and compared using the log-rank test. Multivariate survival analysis was evaluated using Cox’s proportional hazard models to identify independent prognostic factors of disease progression with forward, backward, and stepwise selection of individual factors. All results were considered statistically significant when two-sided p-values were less than 0.05. All analyses were performed by a medical statistician (AY, Ph.D.) using STATA (release 9.2, STATA Inc., College Station, TX, USA).

## Results

### Patient demographics

The median age of all patients was 66 years (range, 44–89 years). The observed tumors were of acinar type adenocarcinoma. The median follow-up period was 44 months (range, 12–154 months) with a median BCR-free survival of 32.0 months. A summary of clinicopathological characteristics is shown in Table 1.

**Table 1:** Summary of clinico-pathologic characteristics (N = 613)

Parameter	Number (%)
Age (Median, range; years)	66 (44–89)
Initial PSA level (Median, range; ng/dL)	8.0 (1–79)
Gleason score ≤6	342 (55.8)
3+4	128 (20.9)
4+3	74 (12.1)
≥8	79 (12.9)
Tumor multiplicity single	192 (31.3)
multiple	421 (68.7)
Extra-prostatic capsule extension	205 (33.4)
Positive surgical margin	154 (25.1)
Positive lymphovascular invasion	50 (8.2)
Positive perineural invasion	340 (55.5)
Seminal vesicle invasion	75 (12.2)
High grade PIN	352 (57.4)
pTstage by AJCC 7^th^. Edition*	
pT2	405 (65.0)
pT3	177 (34.5)
pT4	1 (0.5)
pN+	28 (5.8)
Biochemical recurrence	132 (21.5)
Median follow-up duration (months)	44.0 (12–154)

*, Edge SB, Byrd DR, Compton CC, et al, eds. AJCC Cancer Staging Manual. 7th ed. American Joint Committee on Cancer. Chicago: Springer-Verlag; 2010.

### ERG expression and PTEN loss in prostatic adenocarcinoma

**Fig 1:**
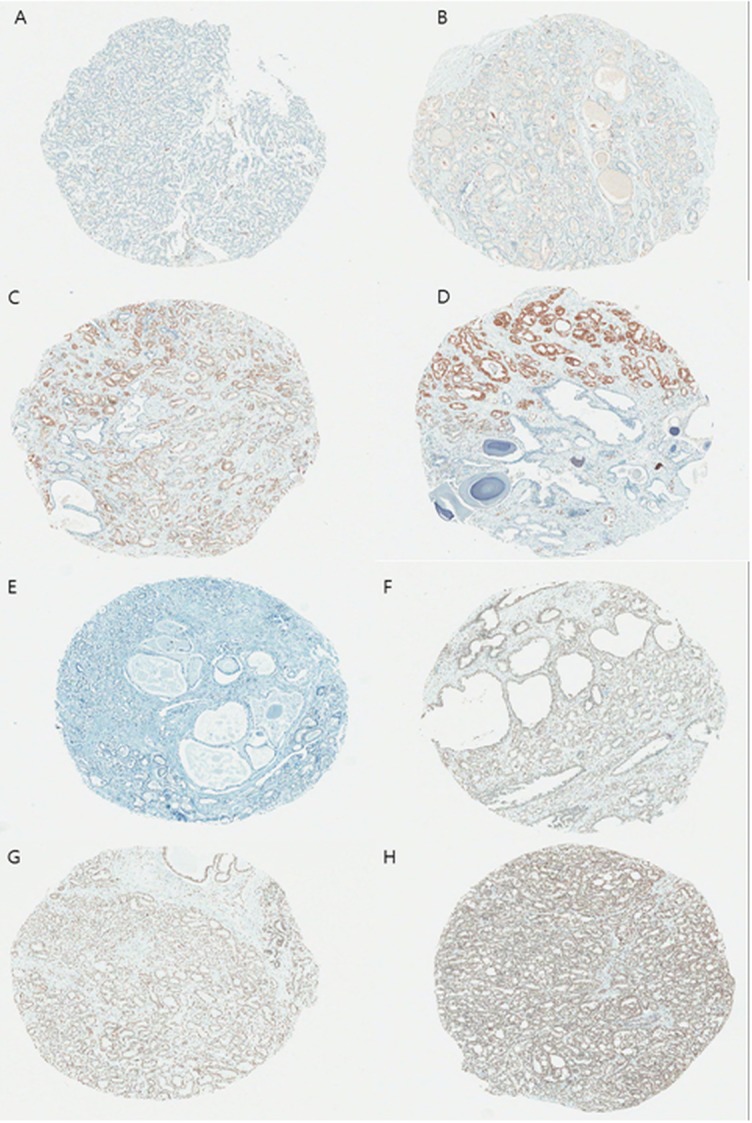
Representative immunohistochemistry for ERG and PTEN in prostate cancer. (A) ERG negative, (B) weak, (C) moderate, and (D) strong, and (E) PTEN negative, (F) weak, (G) moderate, and (H) strong, respectively. (x40).

A summary of the IHC results is shown in Table 2. ERG expression was detected in the nucleus of tumor cells but not in paired normal prostatic tissues (Fig 1A–1D), whereas PTEN expression was observed in the cytoplasm and nucleus of tumor cells and in normal prostatic glands (Fig 1E–1H). ERG positive expression was detected in 145 (23.7%) cases, and absence of PTEN expression, regarded as PTEN loss, was observed in 253 (41.3%) cases.

**Table 2:** Immunohistochemistry results and biochemical recurrence rate for ERG and PTEN expression (N = 613).

Immuno-marker	Number (%)	Number of biochemical recurrence (%)
ERG Positive	145 (23.7)	26 (17.9)
Negative	468 (76.3)	144 (30.8)
PTEN wild type	358 (58.4)	92 (25.7)
loss type	253 (41.3)	78 (12.7)
ERG/PTEN profile		
ERG+/PTEN wild	93 (15.1)	12 (12.9)
ERG+/PTEN loss	52 (8.5)	14 (26.9)
ERG-/PTEN wild	267 (43.6)	78 (29.2)
ERG-/PTEN loss	201 (32.8)	66 (32.8)

### Correlation of ERG expression and PTEN loss with clinicopathological parameters

Pearson’s λ^2^ test or Fisher’s exact test was performed to evaluate the correlation between ERG and PTEN immunostaining and clinicopathological parameters. ERG overexpression had a significant association with patient age (relative risk [RR] = 18.207), initial PSA level (RR = 4.3), Gleason score (RR = 39.262), perineural invasion (RR = 5.17), high-grade prostatic intraepithelial neoplasm (RR = 12.501), and BCR (RR = 8.964) (p<0.05). PTEN loss showed significant correlation with positive surgical margin (RR = 8.524), lymphovascular invasion (RR = 23.445), perineural invasion (RR = 24.489), and pathologic N stage (RR = 11.495) as compared to wild-type PTEN (p<0.05, Table 3).

**Table 3:** Correlation analysis of clinicopathological parameters to ERG and PTEN expression.

	*p* value	
Parameters	ERG expression	PTEN loss
Age (yr) (<60, ≥60)	0.006,RR18.207	0.827
Initial PSA level (ng/mL) (<10, ≥10)	0.039, RR4.300	0.244
Gleason score (≤6, 3+4, 4+3, ≥8)	<0.001,RR39.262	0.095
Tumor volume (<10, ≥10)	0.055	0.078
Surgical margin (Positive, Negative)	0.875	0.033,RR8.524
Extraprostatic extension* (Positive, negative)	0.344	0.977
Lymphovascular invasion (Positive, Negative)	0.332	<0.001,RR23.445
Perienural invasion (Positive, Negative)	0.025, RR5.170	<0.001,RR24.489
Seminal vesicle invasion (Positive, Negative)	0.321	0.369
High grade PIN (Present, Absent)	<0.001,RR12.501	0.382
pTstage (pT2, pT3)	0.488	0.782
pNstage(Positive, Negative)	0.787	0.003,RR11.495
Biochemical recurrence(Present, Absent)	0.002, RR8.964	0.163

*, Extraprostatic capsule extension

### BCR-free survival analysis

**Fig 2:**
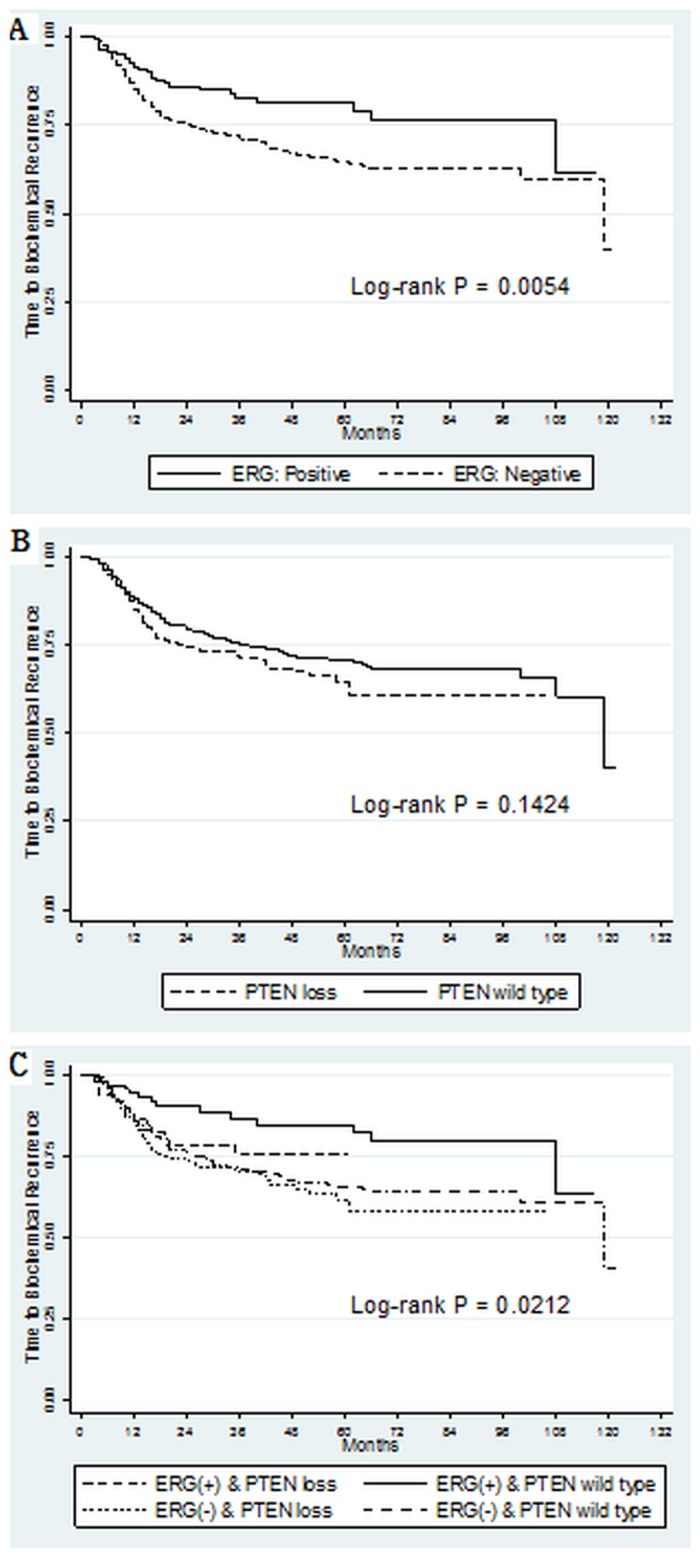
Biochemical recurrence free survival curve according to expression of ERG and PTEN.

At the time of analysis, 132 (21.5%) patients had experienced BCR. Patients with and without ERG expression had significantly different BCR-free survival (p = 0.005, Fig 2A), whereas PTEN loss showed no significant association with BCR-free survival (p = 0.142, Fig 2B). When patients were stratified into four sub-groups according to ERG expression and PTEN loss status, significant differences in BCR-free survival were observed among all groups (p = 0.021, Fig 2C). Those with ERG overexpression and wild-type PTEN had the best BCR-free survival, and patients with ERG negativity and PTEN loss had the worst.

Multivariate analysis identified PSA level (≥10 ng/mL), Gleason score (>6), pathologic T stage (≥T3), positive surgical margin, and extraprostatic capsule extension as significant independent prognostic factors for BCR (p<0.05, Table 4). However, ERG and PTEN profiles were not significant predictive factors for BCR in multivariate analysis (p>0.05) although ERG overexpression was identified as a positive risk factor (hazard ratio [HR] = 0.617, 95% confidence interval [CI] = 0.419–0.908, p = 0.014) and PTEN loss as a negative risk factor (HR = 1.163, 95% CI = 0.863–1.567, p = 0.320) for BCR by univariate analysis (Table 4).

**Table 4:** Association of clinico-pathologic parameters and immunostains with biochemical recurrence free survival based on Cox Proportional Hazards Regression Models.

Variable	Univariate			Multivariable		
	P-value	Hazardratio	95% CI lower –upper limit	P-value	Hazardratio	95% CI lower –upper limit
Age <60	0.519	1.149	0.753–1.754			
≥60, <65	0.213	1.294	0.863–1.941			
≥65, <70	0.898	1.026	0.692–1.522			
≥70						
PSA ≥10	<0.001	2.408	1.775–3.266	0.005	1.612	1.159–2.243
Gleason score ≤6						
3+4	<0.001	2.354	1.579–3.509	0.002	1.907	1.260–2.885
4+3	<0.001	2.875	2.040–4.051	<0.001	2.343	1.631–3.366
≥8	<0.001	5.647	3.820–8.348	<0.001	3.590	2.316–5.563
Tumor volume (≥10%)	<0.001	3.395	2.085–5.527	0.022	1.848	1.092–3.129
Positive Surgical Margin	<0.001	2.318	1.733–3.101	<0.001	1.977	1.467–2.663
Pathologic T stage ≥pT3	<0.001	3.128	2.338–4.185	0.002	8.290	2.249–30.555
Pathologic N stage pN0						
pN1	0.253	1.431	0.774–2.648			
pNx	0.370	0.839	0.572–1.231			
Extraprostatic extension*	<0.001	2.978	2.223–3.989	0.022	0.213	0.057–0.797
ERG overexpression	0.014	0.617	0.419–0.908	0.202	0.768	0.512–1.152
PTEN loss	0.320	1.163	0.863–1.567			

*, Extraprostatic capsule extension

## Discussion

In this study, we evaluated ERG protein expression in PC with reference to normal prostatic tissue using TMAs derived from prostatectomy specimens. IHC was performed instead of fluorescence *in situ* hybridization [9] or quantitative polymerase chain reaction (qPCR), which is widely used to detect ERG gene fusion and its association with known clinicopathological variables for BCR [4, 19, 20]. However, several recent reports have demonstrated the reliability of ERG detection by ERG-specific antibody on paraffin-embedded prostate tissues and the excellent correlation of IHC and FISH for detecting ERG rearrangement [3, 21]. Falzarano *et al*. reported IHC staining of ERG with a high specificity of 99% and sensitivity of 96% in PC specimens obtained from needle biopsy and radical prostatectomy [22, 23]. Furthermore, IHC analysis of ERG expression offers the advantages of a relatively simple, efficient, and low-cost method compared to other molecular techniques used for interpreting ERG fusion expression in prostate specimens [21].

Previous studies have suggested the association between ERG expression and favorable [13] or unfavorable outcomes [4, 8] in PC, which was either in agreement with or contradicting our results. However, loss of PTEN has generally been linked to unfavorable outcomes in PC, which was consistent with the results of this study [4, 11, 13, 24]. Moreover, several prior studies reported no relationship between ERG expression and BCR [17, 25]. The conflicting results on ERG overexpression and its clinical implications might be explained by various reasons, such as the different ethnicity and demographics of enrolled patients and the prognostic endpoint of BCR-free survival, instead of PC-specific mortality, due to short follow-up duration.

Regarding the prognostic value of ERG expression, some studies reported no correlation with disease progression and its associated parameters [25], while others reported a strong prognostic marker [26]. In the present study, we demonstrated that ERG overexpression had a significant impact on BCR-free survival with or without PTEN expression (p = 0.005, Fig 2A) and observed favorable clinical outcome with a HR of 0.768 in multivariate analysis despite statistical insignificance (p = 0.202, Table 4). In addition, the study also showed a high correlation of ERG expression with known prognostic factors such as age, PSA level, Gleason score, extraprostatic capsule extension, and BCR (Table 3). These results were similar to those of previous reports showing that ERG had a prognostic value for PC recurrence, including BCR, showed correlation with Gleason score, and promoted cancer progression in conjunction with PTEN loss [11, 27].

Due to other factors such as ethnic differences and favorable outcome of ERG expression in this study, the proportion of subjects with ERG expression (23.7%) and those with both ERG positivity and PTEN loss (41.3%) differed from previously reported values (ERG, 44–65%; PTEN loss, up to 70%) [19, 25]. The low prevalence of ERG expression and PTEN loss in this study in comparison other studies might be explained by ethnic aspects because other studies of Asian cohorts reported a similar prevalence of ERG and PTEN to ours [21, 28], whereas those conducted in Western population reported higher rates [29, 30]. These ethnic differences have already been suggested as Asian-oriented studies showed a much lower frequency of ERG expression (7.5–28.0%) compared to Caucasian (50.1–52.4%) and African-American (28.2–31.3%) studies [29–32]. In addition, such an ethnic difference might be implicated in the different clinical outcomes of Asian PC patients with ERG overexpression compared to Western patients [2, 28, 32–35]. This meant that PC prevalence and its prognosis, as well as genomic alterations of ERG expression might vary in different geographic locations and according to ethnic differences with the greatest prevalence in Caucasians and the lowest among Asians [36, 37], possibly as a result of specific environmental and/or genetic risk factors affecting Western and Asian men.

In this study of PC specimens, ERG overexpression showed a significant association with patient age (RR = 18.207), initial PSA level (RR = 4.3), Gleason score (RR = 39.262), perineural invasion (RR = 5.17), high-grade prostatic intraepithelial neoplasm (RR = 12.501), and BCR (RR = 8.964) (p<0.05), whereas PTEN loss exhibited a significant correlation with positive surgical margin (RR = 8.524), lymphovascular invasion (RR = 23.445), perineural invasion (RR = 24.489), and pathologic N stage (RR = 11.495) (p<0.05, Table 3). These correlations might be reflected in the prognostic results of combined ERG expression and PTEN loss. ERG overexpression was associated with favorable outcome, hence the correlation with younger patients who often had a lower Gleason score, resulting in a low BCR rate. On the other hand, PTEN loss showed invasive characteristics of PC, such as positive margin and lymphovascular, nodal, and perineural invasion, resulting in unfavorable outcome. However, in the analysis of prognostic factors for BCR, only PSA level (≥10 ng/dL), Gleason score (>6), pathologic T3 stage (≥T3), positive surgical margin, and extraprostatic capsule extension were statistically significant (p<0.05, Table 4), while ERG overexpression and PTEN loss exhibited a positive and negative trend, respectively, without statistical significance (HR = 0.768 and HR = 1.315, respectively) (p<0.05). These parameters with ERG expression [11, 38] and PTEN loss [4, 13, 24] have been shown with similar trends of association with disease progression in previous studies.

In the comparison between PTEN loss and wild-type PTEN, wild-type PTEN was associated with a better BCR-free survival rate, but the result was not statistically significant (p = 0.142, Fig 2B). However, it might suggest potential differences in survival, which could be clarified in future studies that include larger numbers of patients (Fig 2B), because other reports have indicated the prognostic significance of PTEN loss in disease progression [14, 38–41]. Regarding the BCR free-survival analysis of combined PTEN and ERG expression, ERG expression with wild-type PTEN showed the best BCR free-survival among four subgroups (p = 0.016, Fig 2C), suggesting a critical function of ERG with PTEN in PC and that their prognostic association might be stronger when multiplexed with each other, possibly owing to the involvement of a critical pathway of prostate carcinogenesis [11, 13, 17]. Al Bashir *et al*. suggested that the presence of distinct molecular alterations such as CRISP3 (cysteine-rich secretory protein 3) gene in the subgroup of PC with PTEN and ERG expression might have additional clinical implication if they were assessed collectively [13].

The limitation of this study was its retrospective design and short follow-up duration, insufficient for the evaluation of PC-specific mortality. The low rate of ERG and PTEN expression might be speculated as the results of the shrinkage of prostate specimen during pathological specimen processing, different methods of detection and scoring of ERG expression, or technical and material differences in the antibodies used because there is no validated antibody for determining the status both ERG and PTEN. Currently, there are variations in the methods used for identification of ERG rearrangements and in the recording and scoring of expression levels. A consensus must be reached with regard to the clinical utility of ERG prior to its widespread adoption into clinical practice.

## Conclusion

This study shows that ERG expression had predictive values for BCR free survival of PC after radical prostatectomy with initial PSA and pathologic T stage. In addition, the combination of PTEN wild type with ERG positive expression also shows clinical significance in better BCR free survival.

## References

[1] Ferlay J, Shin HR, Bray F, Forman D, Mathers C, Parkin DM. Estimates of worldwide burden of cancer in 2008: GLOBOCAN 2008. International journal of cancer Journal international du cancer. 2010 12 15;127(12):2893–917. Epub 2011/02/26. eng. 10.1002/ijc.25516 21351269

[2] Rubin MA, Maher CA, Chinnaiyan AM. Common gene rearrangements in prostate cancer. Journal of clinical oncology: official journal of the American Society of Clinical Oncology. 2011 9 20;29(27):3659–68. . Epub 2011/08/24. eng. 21859993 10.1200/JCO.2011.35.1916 PMC4874145

[3] Tomlins SA, Palanisamy N, Siddiqui J, Chinnaiyan AM, Kunju LP. Antibody-based detection of ERG rearrangements in prostate core biopsies, including diagnostically challenging cases: ERG staining in prostate core biopsies. Archives of pathology & laboratory medicine. 2012 8;136(8):935–46. . Pubmed Central PMCID: PMC3667408. Epub 2012/08/02. eng. 22849743 10.5858/arpa.2011-0424-OA PMC3667408

[4] Nagle RB, Algotar AM, Cortez CC, Smith K, Jones C, Sathyanarayana UG, et al ERG overexpression and PTEN status predict capsular penetration in prostate carcinoma. The Prostate. 2013 8;73(11):1233–40. Pubmed Central PMCID: PMC4038303. Epub 2013/05/09. eng. 10.1002/pros.22675 23653096 PMC4038303

[5] Carver BS, Tran J, Chen Z, Carracedo-Perez A, Alimonti A, Nardella C, et al ETS rearrangements and prostate cancer initiation. Nature. 2009 2 12;457(7231):E1; discussion E2-3. Pubmed Central PMCID: 2967456. Epub 2009/02/13. eng. 10.1038/nature07738 19212347 PMC2967456

[6] Perner S, Mosquera JM, Demichelis F, Hofer MD, Paris PL, Simko J, et al TMPRSS2-ERG fusion prostate cancer: an early molecular event associated with invasion. The American journal of surgical pathology. 2007 6;31(6):882–8. . Epub 2007/05/29. eng. 17527075 10.1097/01.pas.0000213424.38503.aa

[7] Saramaki OR, Harjula AE, Martikainen PM, Vessella RL, Tammela TL, Visakorpi T. TMPRSS2:ERG fusion identifies a subgroup of prostate cancers with a favorable prognosis. Clinical cancer research: an official journal of the American Association for Cancer Research. 2008 6 1;14(11):3395–400. Epub 2008/06/04. eng. 10.1158/1078-0432.CCR-07-2051 18519769

[8] Demichelis F, Fall K, Perner S, Andren O, Schmidt F, Setlur SR, et al TMPRSS2:ERG gene fusion associated with lethal prostate cancer in a watchful waiting cohort. Oncogene. 2007 7 5;26(31):4596–9. . Epub 2007/01/24. eng. 17237811 10.1038/sj.onc.1210237

[9] Attard G, Clark J, Ambroisine L, Fisher G, Kovacs G, Flohr P, et al Duplication of the fusion of TMPRSS2 to ERG sequences identifies fatal human prostate cancer. Oncogene. 2008 1 10;27(3):253–63. . Pubmed Central PMCID: 2646890. Epub 2007/07/20. eng. 17637754 10.1038/sj.onc.1210640 PMC2646890

[10] Petrovics G, Liu A, Shaheduzzaman S, Furusato B, Sun C, Chen Y, et al Frequent overexpression of ETS-related gene-1 (ERG1) in prostate cancer transcriptome. Oncogene. 2005 5 26;24(23):3847–52. . Epub 2005/03/08. eng. 15750627 10.1038/sj.onc.1208518

[11] Krohn A, Diedler T, Burkhardt L, Mayer PS, De Silva C, Meyer-Kornblum M, et al Genomic deletion of PTEN is associated with tumor progression and early PSA recurrence in ERG fusion-positive and fusion-negative prostate cancer. The American journal of pathology. 2012 8;181(2):401–12. Epub 2012/06/19. eng. 10.1016/j.ajpath.2012.04.026 22705054

[12] Grupp K, Hohne TS, Prien K, Hube-Magg C, Tsourlakis MC, Sirma H, et al Reduced CD147 expression is linked to ERG fusion-positive prostate cancers but lacks substantial impact on PSA recurrence in patients treated by radical prostatectomy. Experimental and molecular pathology. 2013 10;95(2):227–34. Epub 2013/08/21. eng. 10.1016/j.yexmp.2013.08.002 23948277

[13] Al Bashir S, Alshalalfa M, Hegazy SA, Dolph M, Donnelly B, Bismar TA. Cysteine- rich secretory protein 3 (CRISP3), ERG and PTEN define a molecular subtype of prostate cancer with implication to patients' prognosis. Journal of hematology & oncology. 2014;7:21 . Pubmed Central PMCID: PMC3975646. Epub 2014/03/13. eng. 24606912 10.1186/1756-8722-7-21 PMC3975646

[14] Carver BS, Tran J, Gopalan A, Chen Z, Shaikh S, Carracedo A, et al Aberrant ERG expression cooperates with loss of PTEN to promote cancer progression in the prostate. Nature genetics. 2009 5;41(5):619–24. Pubmed Central PMCID: 2835150. Epub 2009/04/28. eng. 10.1038/ng.370 19396168 PMC2835150

[15] Epstein JI, Allsbrook WC Jr., Amin MB, Egevad LL. The 2005 International Society of Urological Pathology (ISUP) Consensus Conference on Gleason Grading of Prostatic Carcinoma. The American journal of surgical pathology. 2005 9;29(9):1228–42. . Epub 2005/08/13. eng. 16096414 10.1097/01.pas.0000173646.99337.b1

[16] Vogel UF, Bueltmann BD. Simple, inexpensive, and precise paraffin tissue microarrays constructed with a conventional microcompound table and a drill grinder. American journal of clinical pathology. 2006 9;126(3):342–8. . Epub 2006/08/02. eng. 16880136 10.1309/F2Q38DXN1V1V4GQM

[17] Kim J, Yu J. Interrogating genomic and epigenomic data to understand prostate cancer. Biochimica et biophysica acta. 2012 4;1825(2):186–96. Pubmed Central PMCID: PMC3307852. Epub 2012/01/14. eng. 10.1016/j.bbcan.2011.12.003 22240201 PMC3307852

[18] Cookson MS, Aus G, Burnett AL, Canby-Hagino ED, D'Amico AV, Dmochowski RR, et al Variation in the definition of biochemical recurrence in patients treated for localized prostate cancer: the American Urological Association Prostate Guidelines for Localized Prostate Cancer Update Panel report and recommendations for a standard in the reporting of surgical outcomes. The Journal of urology. 2007 2;177(2):540–5. . Epub 2007/01/16. eng. 17222629 10.1016/j.juro.2006.10.097

[19] Park K, Tomlins SA, Mudaliar KM, Chiu YL, Esgueva R, Mehra R, et al Antibody-based detection of ERG rearrangement-positive prostate cancer. Neoplasia (New York, NY). 2010 7;12(7):590–8. . Pubmed Central PMCID: 2907585. Epub 2010/07/24. eng. 20651988 10.1593/neo.10726 PMC2907585

[20] Chaux A, Albadine R, Toubaji A, Hicks J, Meeker A, Platz EA, et al Immunohistochemistry for ERG expression as a surrogate for TMPRSS2-ERG fusion detection in prostatic adenocarcinomas. The American journal of surgical pathology. 2011 7;35(7):1014–20. Pubmed Central PMCID: PMC3505676. Epub 2011/06/17. eng. 10.1097/PAS.0b013e31821e8761 21677539 PMC3505676

[21] Suh JH, Park JW, Lee C, Moon KC. ERG immunohistochemistry and clinicopathologic characteristics in Korean prostate adenocarcinoma patients. Korean journal of pathology. 2012 10;46(5):423–8. Pubmed Central PMCID: PMC3490118. Epub 2012/11/09. eng. 10.4132/KoreanJPathol.2012.46.5.423 23136568 PMC3490118

[22] van Leenders GJ, Boormans JL, Vissers CJ, Hoogland AM, Bressers AA, Furusato B, et al Antibody EPR3864 is specific for ERG genomic fusions in prostate cancer: implications for pathological practice. Modern pathology: an official journal of the United States and Canadian Academy of Pathology, Inc. 2011 8;24(8):1128–38. . Epub 2011/04/19. eng. 21499236 10.1038/modpathol.2011.65

[23] Falzarano SM, Zhou M, Hernandez AV, Klein EA, Rubin MA, Magi-Galluzzi C. Single focus prostate cancer: pathological features and ERG fusion status. The Journal of urology. 2011 2;185(2):489–94. Epub 2010/12/21. eng. 10.1016/j.juro.2010.09.093 21167530

[24] Antonarakis ES, Keizman D, Zhang Z, Gurel B, Lotan TL, Hicks JL, et al An immunohistochemical signature comprising PTEN, MYC, and Ki67 predicts progression in prostate cancer patients receiving adjuvant docetaxel after prostatectomy. Cancer. 2012 12 15;118(24):6063–71. Pubmed Central PMCID: PMC3572534. Epub 2012/06/08. eng. 10.1002/cncr.27689 22674438 PMC3572534

[25] Hoogland AM, Jenster G, van Weerden WM, Trapman J, van der Kwast T, Roobol MJ, et al ERG immunohistochemistry is not predictive for PSA recurrence, local recurrence or overall survival after radical prostatectomy for prostate cancer. Modern pathology: an official journal of the United States and Canadian Academy of Pathology, Inc. 2012 3;25(3):471–9. . Epub 2011/11/15. eng. 22080055 10.1038/modpathol.2011.176

[26] Spencer ES, Johnston RB, Gordon RR, Lucas JM, Ussakli CH, Hurtado-Coll A, et al Prognostic value of ERG oncoprotein in prostate cancer recurrence and cause-specific mortality. The Prostate. 2013 6;73(9):905–12. Pubmed Central PMCID: 3677047. Epub 2013/01/22. eng. 10.1002/pros.22636 23334893 PMC3677047

[27] Bismar TA, Dolph M, Teng LH, Liu S, Donnelly B. ERG protein expression reflects hormonal treatment response and is associated with Gleason score and prostate cancer specific mortality. European journal of cancer (Oxford, England: 1990). 2012 3;48(4):538–46. . Epub 2012/02/04. eng. 22300588 10.1016/j.ejca.2012.01.001

[28] Kimura T, Furusato B, Miki J, Yamamoto T, Hayashi N, Takahashi H, et al Expression of ERG oncoprotein is associated with a less aggressive tumor phenotype in Japanese prostate cancer patients. Pathology international. 2012 11;62(11):742–8. Epub 2012/11/06. eng. 10.1111/pin.12006 23121605

[29] Magi-Galluzzi C, Tsusuki T, Elson P, Simmerman K, LaFargue C, Esgueva R, et al TMPRSS2-ERG gene fusion prevalence and class are significantly different in prostate cancer of Caucasian, African-American and Japanese patients. The Prostate. 2011 4;71(5):489–97. Epub 2010/09/30. eng. 10.1002/pros.21265 20878952

[30] Mosquera JM, Mehra R, Regan MM, Perner S, Genega EM, Bueti G, et al Prevalence of TMPRSS2-ERG fusion prostate cancer among men undergoing prostate biopsy in the United States. Clinical cancer research: an official journal of the American Association for Cancer Research. 2009 7 15;15(14):4706–11. Pubmed Central PMCID: 3717524. Epub 2009/07/09. eng. 10.1158/1078-0432.CCR-08-2927 19584163 PMC3717524

[31] Mao X, Yu Y, Boyd LK, Ren G, Lin D, Chaplin T, et al Distinct genomic alterations in prostate cancers in Chinese and Western populations suggest alternative pathways of prostate carcinogenesis. Cancer research. 2010 7 1;70(13):5207–12. Pubmed Central PMCID: 2896548. Epub 2010/06/03. eng. 10.1158/0008-5472.CAN-09-4074 20516122 PMC2896548

[32] Minner S, Enodien M, Sirma H, Luebke AM, Krohn A, Mayer PS, et al ERG status is unrelated to PSA recurrence in radically operated prostate cancer in the absence of antihormonal therapy. Clinical cancer research: an official journal of the American Association for Cancer Research. 2011 9 15;17(18):5878–88. Epub 2011/07/28. eng. 10.1158/1078-0432.CCR-11-1251 21791629

[33] Verdu M, Trias I, Roman R, Rodon N, Garcia-Pelaez B, Calvo M, et al ERG expression and prostatic adenocarcinoma. Virchows Archiv: an international journal of pathology. 2013 6;462(6):639–44. Epub 2013/05/25. eng. 10.1007/s00428-013-1415-3 23703293

[34] Sreenath TL, Dobi A, Petrovics G, Srivastava S. Oncogenic activation of ERG: A predominant mechanism in prostate cancer. Journal of carcinogenesis. 2011;10:37 Pubmed Central PMCID: 3263025. Epub 2012/01/27. eng. 10.4103/1477-3163.91122 22279422 PMC3263025

[35] Furusato B, van Leenders GJ, Trapman J, Kimura T, Egawa S, Takahashi H, et al Immunohistochemical ETS-related gene detection in a Japanese prostate cancer cohort: diagnostic use in Japanese prostate cancer patients. Pathology international. 2011 7;61(7):409–14. Epub 2011/06/29. eng. 10.1111/j.1440-1827.2011.02675.x 21707844

[36] Gronberg H. Prostate cancer epidemiology. Lancet. 2003 3 8;361(9360):859–64. . Epub 2003/03/19. eng. 12642065 10.1016/S0140-6736(03)12713-4

[37] Sim HG, Cheng CW. Changing demography of prostate cancer in Asia. European journal of cancer (Oxford, England: 1990). 2005 4;41(6):834–45. . Epub 2005/04/06. eng. 15808953 10.1016/j.ejca.2004.12.033

[38] Yoshimoto M, Joshua AM, Cunha IW, Coudry RA, Fonseca FP, Ludkovski O, et al Absence of TMPRSS2:ERG fusions and PTEN losses in prostate cancer is associated with a favorable outcome. Modern pathology: an official journal of the United States and Canadian Academy of Pathology, Inc. 2008 12;21(12):1451–60. . Epub 2008/05/27. eng. 18500259 10.1038/modpathol.2008.96

[39] Han B, Mehra R, Lonigro RJ, Wang L, Suleman K, Menon A, et al Fluorescence in situ hybridization study shows association of PTEN deletion with ERG rearrangement during prostate cancer progression. Modern pathology: an official journal of the United States and Canadian Academy of Pathology, Inc. 2009 8;22(8):1083–93. . Pubmed Central PMCID: 2760294. Epub 2009/05/02. eng. 19407851 10.1038/modpathol.2009.69 PMC2760294

[40] Leinonen KA, Saramaki OR, Furusato B, Kimura T, Takahashi H, Egawa S, et al Loss of PTEN Is Associated with Aggressive Behavior in ERG-Positive Prostate Cancer. Cancer epidemiology, biomarkers & prevention: a publication of the American Association for Cancer Research, cosponsored by the American Society of Preventive Oncology. 2013 11 27 . Epub 2013/10/03. Eng. 24083995 10.1158/1055-9965.EPI-13-0333-T PMC4086660

[41] Reid AH, Attard G, Brewer D, Miranda S, Riisnaes R, Clark J, et al Novel, gross chromosomal alterations involving PTEN cooperate with allelic loss in prostate cancer. Modern pathology: an official journal of the United States and Canadian Academy of Pathology, Inc. 2012 6;25(6):902–10. . Epub 2012/03/31. eng. 22460813 10.1038/modpathol.2011.207

